# Priority Questions and Horizon Scanning for Conservation: A Comparative Study

**DOI:** 10.1371/journal.pone.0145978

**Published:** 2016-01-27

**Authors:** Salit Kark, William J. Sutherland, Uri Shanas, Keren Klass, Hila Achisar, Tamar Dayan, Yael Gavrieli, Ronit Justo-Hanani, Yael Mandelik, Nir Orion, David Pargament, Michelle Portman, Orna Reisman-Berman, Uriel N. Safriel, Gad Schaffer, Noa Steiner, Israel Tauber, Noam Levin

**Affiliations:** 1 The Biodiversity Research Group, The School of Biological Sciences, ARC Centre of Excellence for Environmental Decisions, The University of Queensland, Brisbane, 4072, Australia; 2 The Biodiversity Research Group, The Hebrew University of Jerusalem, Jerusalem, 91904, Israel; 3 Conservation Science Group, Department of Zoology, University of Cambridge, Cambridge, CB2 3EJ, United Kingdom; 4 Dept. of Biology and Environment, University of Haifa-Oranim, Tivon, 36006, Israel; 5 HaMa’arag – The Israel National Program for Ecosystem Assessment, Israel Academy of Sciences and Humanities, Albert Einstein Square, Jerusalem, 91040, Israel; 6 Dept. of Zoology, Faculty of Life Sciences, Tel-Aviv University, Tel-Aviv, 69978, Israel; 7 Nature Campus, Department of Zoology, Tel Aviv University, Tel Aviv, 69978, Israel; 8 Dept. of Entomology, Hebrew University of Jerusalem, POB 012, Rehovot, 76100, Israel; 9 Earth and Environmental Sciences group, Dept. of Science Teaching, The Weizmann Institute of Science, P.O. Box 26, Rehovot, 76100, Israel; 10 Yarqon River Authority, PO Box 6297, Tel Aviv, 61067, Israel; 11 Faculty of Architecture and Town Planning, Technion - Israel Institute of Technology, Haifa, 32000, Israel; 12 French Associates Institute for Agriculture and Biotechnology of Drylands, Blaustein Institutes for Desert Research, Ben Gurion University of the Negev, Sede Boqer Campus, Sede Boqer, 84990, Israel; 13 Dept. of Ecology, Evolution and Behavior, The Hebrew University of Jerusalem, Jerusalem, 91904, Israel; 14 Dept. of Geography, Faculty of Social Sciences, The Hebrew University of Jerusalem, Mt Scopus, Jerusalem, 91905, Israel; 15 Open Landscapes and Biodiversity Division, The Israel Ministry of Environmental Protection, Jerusalem, Israel; 16 Forest Management, Monitoring and GIS, KKL – Land Development Authority – Forest Department, The Jewish National Fund, Eshtaol, Israel; University of Waikato (National Institute of Water and Atmospheric Research), NEW ZEALAND

## Abstract

Several projects aimed at identifying priority issues for conservation with high relevance to policy have recently been completed in several countries. Two major types of projects have been undertaken, aimed at identifying (i) policy-relevant questions most imperative to conservation and (ii) horizon scanning topics, defined as emerging issues that are expected to have substantial implications for biodiversity conservation and policy in the future. Here, we provide the first overview of the outcomes of biodiversity and conservation-oriented projects recently completed around the world using this framework. We also include the results of the first questions and horizon scanning project completed for a Mediterranean country. Overall, the outcomes of the different projects undertaken (at the global scale, in the UK, US, Canada, Switzerland and in Israel) were strongly correlated in terms of the proportion of questions and/or horizon scanning topics selected when comparing different topic areas. However, some major differences were found across regions. There was large variation among regions in the percentage of proactive (i.e. action and response oriented) versus descriptive (non-response oriented) priority questions and in the emphasis given to socio-political issues. Substantial differences were also found when comparing outcomes of priority questions versus horizon scanning projects undertaken for the same region. For example, issues related to climate change, human demography and marine ecosystems received higher priority as horizon scanning topics, while ecosystem services were more emphasized as current priority questions. We suggest that future initiatives aimed at identifying priority conservation questions and horizon scanning topics should allow simultaneous identification of both current and future priority issues, as presented here for the first time. We propose that further emphasis on social-political issues should be explicitly integrated into future related projects.

## Introduction

In recent years, awareness is increasing as to the importance of improving the links between environmental science, practice and policy [1]. Under this framework, several projects aimed at collaboratively identifying priority questions for biodiversity conservation with high relevance to environmental policy have recently been undertaken. The identification of key questions can assist in better prioritization of future applied research directions and in strengthening the needed ties between academics, policy makers, managers, practitioners and many other stakeholders [1,2], all of whom influence and shape the future of biodiversity conservation. Such efforts can also help in directing future work, research agendas, initiatives, funding and resources in the field. Beginning in the UK [3], results of such processes have been published in the US [4], in Canada [5] and at a global scale [6] and have most recently tackled subjects such as global agriculture [7], food systems [8], Canadian ocean science [9] and fundamental ecology [10]. A related project focusing on ecological continuum maintenance and restoration has recently been completed in the Swiss Alps [11].

### Top questions and horizon scanning issues

Two main types of projects have been completed in the context of biodiversity conservation. The first focuses on identifying important policy-relevant research questions that are most imperative to conservation. The second, termed horizon scanning, aims to identify emerging issues that are currently not emphasized, but are expected to have serious impacts on biodiversity conservation in the future. Horizon scanning projects can be limited to specific regions [12] or taxa [13] and may involve a second stage of assessment to determine actions by practitioners [14]. While each of the projects completed to date had its own unique emphasis, participants and specific framework, the different projects also share many similarities in goals, targets, structure and format.

In this paper, our main endeavor is to compare conservation-oriented projects that are based on priority conservation questions and horizon scanning. Such comparisons can assist in developing further projects, in directing ongoing and future similar efforts worldwide, and can provide a baseline for comparing the factors shaping the translation of the project outcomes to prioritization of research projects. Our aims were to: (1) synthesize the results of the main conservation-oriented questions and horizon scanning projects completed and published around the world between 2006–2011, (2) examine whether different projects similarly prioritize the major issues facing conservation policy today, (3) examine whether outcomes of projects identifying important questions are similar to those of horizon scanning projects and (4) add a Mediterranean case study in addition to the UK, US, Canadian, Swiss and global projects.

### Adding the first Mediterranean case study

To widen the scope of the synthesis across countries and add a novel perspective from a biodiversity hotspot region that has not been the focus of such a project to date, we present the original results of the first national-scale question identification and the first horizon scanning project undertaken in a Mediterranean country (Israel). Israel is a small (22,000 km^2^) and very densely populated [15,16] biodiversity hotspot located in the Eastern Mediterranean at the crossroads of three continents (Europe, Asia and Africa). In this region, humans have been actively exploiting ecosystems for thousands of years (e.g., grazing, traditional agriculture [17,18]). The rate of human population growth in Israel is among the highest in industrial countries, leading to rapid urbanization and severe threats to its natural ecosystems and biodiversity [19]. Israel has exhibited intense development and substantial natural habitat loss since it gained independence in 1948. Its geographical, demographic, socio-political, and ecological set of conditions make Israel an interesting case study and compare with other, far larger countries (e.g., the US, Canada and the UK) where several such exercises have recently been conducted over the past decade.

The goal of the project undertaken in Israel was to identify scientific questions that, if answered, would have the greatest impact on biodiversity conservation in Israel. Simultaneously with the priority questions project, we conducted a horizon scanning exercise within the same framework (and workshop). These were defined as issues and gaps in current research that are likely to have serious impacts on biodiversity conservation in Israel in the future (10–25 years forward), but do not currently receive sufficient attention and should be further examined with some urgency to allow appropriate response in the future. This project, completed in a collaborative effort by Israeli scientists, practitioners, managers and policy makers is to our knowledge the first attempt globally to simultaneously undertake identification of priority questions and horizon scanning topics in the framework of a single project, by the same team and for the same region [20]. In this paper, as part of the global synthesis, we present the outcomes of the project in Israel and include its outcomes and perspectives in the global comparison with the other priority questions and horizon scanning projects completed and published around the world until mid 2015 (in the UK, US, Canada, Switzerland and globally).

### Synthesis of project outcomes

Here, we also aimed to examine whether different projects have different emphases in the selection of priority questions and horizon scanning topics. It is difficult to disentangle the multiple factors shaping the outcomes of specific projects as multiple factors may be affecting the outcomes. In comparison of the outcomes of the two major types of projects (priority questions vs. horizon scanning) we predicted that, in all cases, climate change would be viewed as an important future threat rather than an issue that should be addressed immediately. When including the recent Mediterranean case study, we predicted that compared with the US, Canada, Switzerland and the UK, freshwater-related issues will be more emphasized in Israel, where water is a major limiting factor of biological productivity with over half of the area classified as arid or semi-arid [21] and prone to desertification [22].

We predicted that cross-boundary issues would receive greater attention in Israel due to its location at a crossroads between continents, the political background and long-term conflicts with its neighbors, and also by the relative high ratio of international border length to area. Because climate change and ecosystem services issues have been mostly neglected and received relatively limited attention in Israel until very recently, we predicted that they will receive relatively little attention in the priority questions selected in the project conducted in Israel.

## Materials and Methods

### Comparison of the questions and horizon scanning project outcomes

We compared the results of published top questions and horizon scanning projects that focused on policy-relevant environmental, biodiversity and conservation issues and that were completed between 2006–2014 (see Tables 1 and 2 for the questions projects and horizon scanning projects included, respectively). The original UK project [3], which was the first to adopt this approach to identify questions of importance, had a slightly different emphasis, focusing on ecological questions of high relevance to policy. We included this earlier UK project here to allow reference to the earlier project. For each project, we calculated the total number and percentage of questions/horizon scanning topics that addressed 10 main topic categories we identified in advance, which aimed to cover the majority of conservation-related issues. The following categories were compared across the projects:

1. Climate change: including questions that refer to current or future effects of climate change and related policy, management and climate-adaptation issues.
2. Socio-political issues, active policy: questions that contain a component referring directly to governance, policy or politics.
3. Demographic issues: questions that specifically refer to human population size, growth or human movement (e.g., immigration) or to changing demographics within human populations (e.g., urbanization).
4. Human related systems: questions that refer directly to human-dominated landscapes (including agriculture), human settlements, infrastructure etc.
5. Freshwater systems: questions that explicitly refer to freshwater environments, freshwater use or needs, including questions that referred specifically to freshwater, or to more general issues that mentioned freshwater or water.
6. Marine systems: questions explicitly referring to the marine environment in any way, such as its biodiversity, threats, infrastructure, and protected areas.
7. Ecosystem services: questions that make specific reference to benefits that people gain from ecosystems, to the phrase ecosystem services itself, or to the economic value of natural resources.
8. Cross-boundary issues: questions that deal directly with cross-boundary and/or border-related issues, cooperation amongst different countries, global solutions to conservation problems, and to the effect of cross-boundary conflict on biodiversity.
9. Descriptive: questions that describe a problem or threat, without referring to past, present or future solutions or responses to that problem or threat.
10. Proactive: questions that are action and response oriented, and deal with, or propose, solutions and/or actions aimed to address a problem or threat. We included in this category questions that addressed the effectiveness of policies or management approaches and practices and questions that suggest which management practices and policies best address a particular issue.

**Table 1:** The number and percentage of priority questions that deal with each of the major categories examined in the projects. These include: climate change; socio-political issues or policy; human demographic issues (population size etc.); human related systems (including agriculture, human settlements, human-dominated landscapes, infrastructure etc.); freshwater systems (e.g., freshwater, rivers, water market etc.); marine systems; ecosystem services; cross-boundary issues (political boundaries, neighboring countries etc.); descriptive questions: those that describe and study problem/threat; proactive questions: questions that deal with solutions and action to address problem. The table provides the number and percentage of questions in each category. A question can assigned to none, a single or more than one category.

	Israel questions^1^	UK (2006) questions^2^	UK (2010) questions^3^	US (2011) questions^4^	Canada (2011) questions^5^	Global (2009) questions^6^	Switzerland (2012) questions^7^	US (2014) questions^8^
Focus of project	Biodiversity conservation	Policy-relevant ecology Qs	Conservation policy	Conservation science and policy	Conservation and resource management policy	Biodiversity conservation	Action-oriented conservation science	Resource management policy
Total number of selected questions	45	100	69	40	40	100	44	40
Climate change	2 (4.4%)	11 (11%)	14 (20.3%)	11 (27.5%)	3 (7.5%)	20 (20%)	3 (6.8%)	12 (30%)
Socio-political issues	11 (24.4%)	6 (6%)	15 (21.7%)	9 (22.5%)	16 (40%)	26 (26%)	5 (11.4%)	5 (12.5%)
Human demography	1 (2.2%)	0 (0%)	0 (0%)	2 (5%)	3 (7.5%)	7 (7%)	0 (0%)	2 (5%)
Human related systems	15 (33.3%)	34 (34%)	15 (21.7%)	20 (50%)	6 (15%)	27 (27%)	8 (18.2%)	21 (52.5%)
Freshwater systems	4 (8.9%)	15 (15%)	18 (26.1%)	3 (7.5%)	8 (20%)	8 (8%)	2 (2.3%)	4 (10%)
Marine systems	5 (11%)	17 (17%)	5 (7.2%)	7 (17.5%)	3 (7.5%)	11 (11%)	0 (0%)	5 (12%)
Ecosystem services	5 (11%)	4 (4%)	12 (17.4%)	5 (12.5%)	7 (17.5%)	18 (18%)	0 (0%)	4 (10%)
Cross-boundary issues	4 (8.9%)	1 (1%)	0	3 (7.5%)	1 (2.5%)	8 (8%)	0 (0%)	1 (2.5%)
Descriptive: describe and study problem/threat	17 (37.8%)	52 (52%)	26 (37.7%)	27 (67.5%)	17 (42.5%)	41 (41%)	18 (40.9%)	33 (82.5%)
Proactive: deal with solutions and action to address problem	34 (75.6%)	45 (45%)	42 (60.9%)	9 (22.5%)	21 (52.5%)	57 (57%)	26 (59.1%)	9 (22.5%)

Sources (Table 1):

^1^. This paper.

^2^. Sutherland WJ, Armstrong-Brown S, Armsworth PR, Brereton T, Brickland J, Campbell CD, et al. (2006) The identification of 100 ecological questions of high policy relevance in the UK. J Appl Ecol 43: 617–627.

^3^. Sutherland WJ, Albon SD, Allison H, Armstrong-Brown S, Bailey MJ, et al. 2010. The identification of priority policy options for UK nature conservation. Journal of Applied Ecology 47: 955–965.

^4^. Fleishman E, Blockstein DE, Hall JA, Mascia MB, Rudd MA, Scott JM, et al. (2011) Top 40 priorities for science to inform US conservation and management policy. Bioscience 61: 290–300.

^5^. Rudd MA, Beazley KF, Cooke SJ, Fleishman E, Lane DE, Mascia MB, et al. (2011) Generation of priority research questions to inform conservation policy and management at a national level. Conserv Biol 25: 476–484.

^6^. Sutherland WJ, Adams WM, Aronson RB, Aveling R, Blackburn TM, Broad G, et al. (2009) One hundred questions of importance to the conservation of global biological diversity. Conserv Biol 23: 557–567.

^7^. Braunisch V, Home R, Pellet J, Arlettaz R. (2012) Conservation science relevant to action: A research agenda identified and prioritized by practitioners. Biological Conservation 153: 201–210.

^**8**^. Rudd MA, Fleishman E. (2014) Policymakers’ and scientists’ ranks of research priorities for resource-management policy. Bioscience 1–10.

**Table 2:** The number and percentage horizon scanning topics that deal with the following topics or issues: climate change; socio-political issues or policy; human demographic issues (population size etc.); human related systems (including agriculture, human settlements, human-dominated landscapes, infrastructure etc.); freshwater systems (e.g., freshwater, rivers, water market etc.); marine systems; ecosystem services; cross-boundary issues (political boundaries, neighboring countries etc.); descriptive questions: those that describe and study problem/threat; proactive questions: questions that deal with solutions and action to address problem. The table provides the number and percentage of horizon scanning topics in each category. A horizon scanning topic can belong to none, a single or more than one category.

	Israel horizon scanning^1^	UK (2008) horizon scanning^2^	Global (2010) horizon scanning ^3^	Global (2011) horizon scanning^4^	Global (2012) horizon scanning^5^	Global (2013) horizon scanning^6^	Global (2014) horizon scanning^7^
Focus of project	Biodiversity conservation	Biodiversity conservation	Biodiversity conservation	Biodiversity conservation	Biodiversity conservation	Biodiversity conservation	Biodiversity conservation
Number of topics selected	8	25	15	15	15	15	15
Climate change	2 (25%)	11 (44%)	7 (46.7%)	6 (40%)	5 (33.3%)	3 (20%)	4 (26.7%)
Socio-political issues	3 (37.55%)	10 (40%)	1 (6.7%)	3 (20%)	1 (6.7%)	0 (0%)	5 (33.3%)
Human demography	1 (12.5%)	2 (8%)	0 (0%)	1 (6.7%)	1 (6.7%)	0 (0%)	0 (0%)
Human related systems	5 (62.5%)	7 (28%)	6 (40%)	6 (40%)	8 (53.3%)	7 (46.7%)	6 (40%)
Freshwater systems	1 (12.5%)	5 (20%)	4 (26.7%)	4 (26.7%)	4 (26.7%)	4 (26.7%)	2 (13.3%)
Marine systems	3 (37.5%)	7 (28%)	8 (53.3%)	4 (26.7%)	6 (40%)	4 (26.7%)	4 (26.7%)
Ecosystem services	0 (0%)	3 (12%)	0 (0%)	0 (0%)	0 (0%)	1 (6.7%)	0 (0%)
Cross-boundary issues	1 (12.5%)	1 (4%)	1 (6.7%)	1 (6.7%)	0 (0%)	1 (6.7%)	3 (20%)
Descriptive: describe and study problem/threat	4 (50%)	21 (84%)	12 (80%)	13 (86.7%)	9 (60%)	5 (33.3%)	7 (46.7%)
Proactive: deal with solutions/actions to address problem	4 (50%)	8 (32%)	6 (40%)	2 (13.3%)	7 (46.7%)	10 (66.7%)	9 (60%)

Sources (Table 2):

^1^. This paper.

^2^. Sutherland WJ, Bailey MJ, Bainbridge IP, Brereton T, Dick JTA, Drewitt J, et al. (2008) Future novel threats and opportunities facing UK biodiversity identified by horizon scanning. J Appl Ecol 45: 821–833.

^3^. Sutherland WJ, Clout M, Cote IM, Daszak P, Depledge MH, Fellman L, et al. (2010) A horizon scan of global conservation issues for 2010. Trends Ecol Evol 25: 1–7.

^4^. Sutherland WJ, Bardsley S, Bennun L, Clout M, CôtéIM, Depledge MH, et al. (2011) Horizon scan of global conservation issues for 2011. Trends Ecol Evol 26: 10–16.

^5^. Sutherland WJ, Aveling R, Bennun L, Chapman E, Clout M, Côté IM, et al. (2012) A horizon scan of global conservation issues for 2012. Trends Ecol Evol 27(1): 12–18.

^6^. Sutherland WJ, Bardsley S, Clout M, Depledge MH, Dicks LV, Fellman L, et al. (2013) A horizon scan of global conservation issues for 2013. Trends Ecol Evol 28(1): 16–22.

^7^. Sutherland WJ, Aveling R, Brooks TM, Clout M, Dicks LV, Fellman L, et al. (2014) A horizon scan of global conservation issues for 2014. Trends Ecol Evol 29(1): 15–22.

Questions were considered “proactive” if they explicitly addressed current solutions or actions, proposed future solutions or actions, or raised the question of how issues may be practically addressed, whereas “descriptive” questions were those that included no reference to a solution or policy/management response to the issue at hand, but rather focused only on the components of the issue itself. The key to distinguishing between the two was whether management/planning/policy alternatives/outcomes/effects were explicitly referred to. The following examples from the categorization of the questions from the Israeli exercise illustrate this distinction: the question “How do different management actions influence the resilience and rehabilitation dynamics of biodiversity in different habitats in Israel following fire events?” was categorized as proactive, as it only refers to the outcomes of practical management decisions, while the question “What are the impacts of various synthetic materials and compounds (such as estrogen analogs, nano-particles, TBT, synthetic nitrogen and pesticides) on biodiversity, and how can these negative impacts be minimized?” is both proactive and descriptive, as the first half refers to ecological and biological processes, whereas the second half refers to potential management/policy actions that might mitigate the effects of these processes. In order to minimize ambiguity, the classification of questions in all projects included was done by the project leaders, and was based solely on each question as it was phrased, rather than, for example, inference of additional possible relevance to other fields or general knowledge of the project leaders (SK, NL, US). Unless clearly stated otherwise in the text of the question, all questions were understood to refer to the geographical extent defined by the paper’s scope (e.g. USA, Canada, UK, Switzerland), and not to broader cross-boundary scales.

Given the complexity of many of the questions, we assigned more than one category to a question where it was merited, but based these categorizations on the specific components included in each question. We therefore included in the marine category, for example, only those questions that specifically included the words marine, ocean, coastal, reference to industries that were stated to explicitly impact the marine ecosystem (e.g. desalinization plants, natural gas extraction in the Mediterranean etc.)m specific marine ecosystem services or biodiversity components. Broad questions regarding, for example, patterns of biodiversity were not assigned to the marine systems category unless this ecosystem was explicitly included in the question. Thus, for example, the question “How can the effects of intense anthropogenic activity (such as construction, light, noise pollution and quarries) on biodiversity components in Israel and their functioning in built-up and disturbed areas be mitigated or eliminated?” formulated in the Israeli project was assigned to category #4 and category #10, but not categories #5 or #6.

Following this, we generated a correlation matrix using Pearson's correlation coefficient between the proportions of the priority questions per category identified in each pair of projects that focused on identifying priority questions (see Table 3). The same was done for the horizon scanning projects (Table 4). We also generated a table presenting the proportion of selected questions and that of the priority horizon scanning topics per category for each project separately (Table 5). This was done for each region that had completed both questions and horizon scanning projects (Israel, UK, and the global project).

**Table 3:** Pearson's correlation coefficients between the proportion that each category received in the final top list of questions in each of the projects included in this study (see Table 1).

Variables	UK (2006) ecology questions	UK (2010) questions	US (2011) questions	Canada (2011) questions	Global (2009) questions	Switzerland (2012) questions	US (2014) questions
Israel questions	0.805 **	0.880 ***	0.451	0.835 **	0.944 ***	0.952 ***	0.422
UK (2006) ecology questions		0.794 **	0.791 **	0.670 *	0.822 **	0.877 ***	0.812 **
UK (2010) questions			0.446	0.872 ***	0.909 ***	0.905 ***	0.463
US (2011) questions				0.429	0.594	0.575	0.982 ***
Canada (2011) questions					0.867 **	0.846 **	0.407
Global (2009) questions						0.968 ***	0.570
Switzerland (2012) questions							0.582

Significance levels:

* p < 0.05,

** p < 0.01,

*** p < 0.001

**Table 4:** Pearson's correlation coefficients between the proportion that each category received in the final top horizon scanning topics for each of the projects included in this study and shown in Table 2.

Variables	UK (2008) horizon scanning	Global (2010) horizon scanning	Global (2011) horizon scanning	Global (2012) horizon scanning	Global (2013) horizon scanning	Global (2014) horizon scanning
Israel horizon scanning	0.635	0.674 *	0.708 *	0.803 **	0.700 *	0.903 ***
UK (2008) horizon scanning		0.812 **	0.930 ***	0.714 *	0.441	0.775 *
Global (2010) horizon scanning			0.916 ***	0.933 ***	0.774 *	0.746 *
Global (2011) horizon scanning				0.878 **	0.683 *	0.805 **
Global (2012) horizon scanning					0.912 ***	0.750 *
Global (2013) horizon scanning						0.624

P values:

* p < 0.05,

** p < 0.01,

*** p < 0.001

**Table 5:** The ratio (expressed in percentage) between the proportion of top questions within each category, divided by its proportion in a horizon scanning project completed in the same region, calculated for three regions where both questions and horizon scanning projects were completed (Israel, UK and globally). Equal values between the percentage of questions and horizon scanning results compared per category in each region are 100%. Values below 100% indicate that a category was more frequent in the horizon scanning than in its respective priority questions. Where more than one questions project or horizon scanning project was done for the same region (UK questions and global horizon scanning), the publication year is marked in parentheses.

**Category**	**Region**				
	Israel	UK		Global	
	Horizon Scanning (this paper)	Horizon Scanning (2008)	Horizon Scanning (2008)	Horizon Scanning (2010)	Horizon Scanning (2011)
	Questions	Questions (2006)	Questions (2010)	Questions (2009)	Questions (2009)
Climate change	18%	25%	46%	43%	50%
Socio-political	65%	15%	54%	388%	130%
Human demography	18%	0%	0%	*	104%
Human related systems	53%	121%	78%	68%	68%
Freshwater systems	71%	75%	131%	30%	30%
Marine systems	29%	61%	26%	21%	41%
Ecosystem services	*	33%	145%	*	*
Cross-boundary	71%	25%	0%	119%	119%
Descriptive	76%	62%	45%	51%	47%
Proactive	151%	141%	190%	143%	429%

* An asterisk indicates a case where the percentage of a specified category was zero for the HS exercise but >0 in the questions project for the same region.

To compare the proportion of questions per category identified in each of the projects, we calculated the Odds Ratio, a measure often used by epidemiologists, which compares whether the probability of a certain event is the same for two groups [23,24]. The Odds Ratio has been proposed as providing powerful support to ecological interpretations based on the comparison of proportions [23]. Further details on the methods of the Israel questions and horizon scanning projects included in this paper are provided below and in S1 and S2 Appendices. We did not obtain ethics approval for this exercise, as it was agreed from the outset that those participating in the voting and selection of questions were to become authors of the resulting paper. All submitted questions were treated anonymously. All studies included in this paper were undertaken before the priority questions and horizon scanning exercise in Israel and details of the methods used are available in published papers (see reference list). We provide the details for the Israel project method below, as this is the first time they are published.

### Detailed Methods for the priority questions and horizon scanning project undertaken in Israel

#### A Mediterranean perspective: The Israel project

The priority questions and horizon scanning project presented in this paper was the first such project to be performed in Israel and, as far as we are aware, it was also the first such exercise to be held in any Mediterranean or Middle Eastern country, aiming to prioritize the priority research questions and horizon scanning topics in a wide participatory process. It was also the first exercise globally to perform both priority questions identification and horizon scanning in one framework, at the same workshop and with the same forum of participants. The questions and horizon scanning project took place in Israel between September 2010 and December 2011 and was comprised of the following main steps:

#### Planning phase

The first phase of the project included meetings and a one-day workshop in which the format of the questionnaire to be sent out, the number of questions, and the spatial extent of the project (terrestrial, marine, aquatic) were defined. The workshop included approximately 20 invited experts from a range of organizations in Israel (S1 Appendix) and two invited international guests (William Sutherland from the University of Cambridge, who has led previous related projects in the UK, globally and elsewhere, and advised in this project, and Dr. Xavier La Roux of the French Foundation for Biodiversity, both whom participated in the first planning workshop).

#### Submission of questions and horizon scanning topics

We aimed to undertake a wide-participatory process and receive input from a wide range of governmental, non-governmental and research organizations (S1 Appendix). Therefore, we both identified representatives from a large range of relevant organizations in Israel and solicited questions and topics, mostly through an Internet website specifically constructed for this purpose. We also created a list of representatives in each of the participating organizations to take part in planning the questionnaire format, distributing the questionnaire (in Hebrew) among their organization members and attending the final workshop (see below). In addition, during the first six months of the project, email, phone, printed and web invitations were sent to thousands of people from dozens of organizations in Israel who work in the area of biodiversity, conservation, natural resource management or in related fields, inviting them to submit questions via the project website. The question submission website was also advertised at conference talks and presentations, via brochures, flyers, and through hundreds of individual emails and personal phone calls by the project leading team to stakeholders and others with an interest in the environment and nature conservation. Questions and horizon scanning topics were then submitted, mostly in Hebrew, using the dedicated Internet website. Questions were summited by included a wide range of representatives from relevant organizations, such as academic institutions in Israel, funding agencies, government agencies, non-governmental organizations and others (see list in S1 Appendix).

The most useful mode of advertisement for achieving responses (as estimated by the number of web page visits and questions submitted) was via plenary talks at large workshops and conferences where the project leaders described the project and invited experts to submit questions by providing the project web address. Based on Google Statistics data we assessed the number of people visiting the web page and found that immediately following a talk at a conference that included information about the project and the web link, the number of submitted questions increased dramatically (the peak was usually on the day of the conference talk and the following day).

The on-line questionnaire was divided into two main sections, starting with the questions submission and followed by the horizon scanning topic submission (the latter was optional). In the priority questions project, submitters were asked (in Hebrew) to “identify policy-relevant scientific questions that, if answered, would have the greatest impact on biodiversity conservation in Israel”. In the horizon scanning section, submitters were asked to “identify the most critical horizon scanning topics: future (10–25 years) issues and gaps in current research that are likely to have serious impacts on biodiversity conservation but do not currently receive sufficient scientific attention.”

Overall, 99 different people submitted 238 questions for Israel. Of these, the 90 non-anonymous submitters included 29 with PhD degrees, three PhD students, 28 with a MA or MSc degree and 12 people with a BA or BSc degree. Eighteen participants either did not have an academic degree or did not provide this information. Submitters who specified their affiliation came from 30 different organizations—academia, government agencies and ministries, non-governmental organizations (NGOs), private consultancy and touring companies, among others. People who submitted questions on-line came from 22 different professional backgrounds based on their own expertise selection in the web page. These included: nature and biodiversity conservation, geography, conservation enforcement (rangers), nature and travel guiding, environmental science, ecology, ecology of aquatic systems, marine biology, zoology, policy, planning, management, forestry and land management, environmental consulting, organic farming, economics, engineering, foreign trade administration, law, sociology, environmental philanthropy and tourism.

#### Sorting of questions prior to the final workshop

The project leaders reviewed all the questions submitted electronically to the project website between 1^st^ March and 14^th^ May 2011. After removing duplicate entries (some questions were submitted twice), and separating single questions that were composed of several different sub-questions, there remained a list of 238 research questions and 35 horizon scanning questions. We examined the content of each question carefully to ensure they comply with the basic instructions and removed 4 of the 238 questions submitted via the website that had no direct or indirect connection to biodiversity, habitat or ecosystem service conservation planning, policy, science or management, and were thus clearly irrelevant. One horizon scanning question was removed by the project leaders, as it clearly did not fit the criteria for a horizon scanning topic. This left a total of 234 research questions and 34 horizon scanning topics.

We then carefully reviewed publications from similar projects completed in other countries and globally (see references for Tables 1 and 2) and adapted questions that were relevant to conservation in Israel and referred to topics that had been either unaddressed or only partially addressed in the questions submitted online. This process added 60 questions to the list of candidate research questions submitted online. This included 23 questions adapted from Sutherland et al. 2009 (top 100 questions for global conservation), 25 questions adapted from Sutherland et al. 2006 (top 100 ecology questions for UK) and 8 questions adapted from Rudd et al. 2011 (top 40 conservation questions for Canada). Eleven additional research questions were added after completing this process by the workshop organizers, bringing the total to 301. One horizon scanning topic was added by the workshop organizers, bringing the total to 35 topics. All questions and horizon scanning topics were categorized into 10 general categories determined by the project leaders and workshop organizers (SK, NL and US), corresponding to the topics to be covered by the subgroups at the workshop (see below).

#### Question and topic selection workshop—participants and process

Following lessons learned from the process in other countries, we convened a two-day workshop, in which we distilled and created a shortlist of the major policy-relevant research questions for conservation in Israel based on the above steps. During the workshop, participants voted on the priority questions to be included in the final list. See details below and S1 Appendix for the list of organizations represented in the final workshop.

The project leaders (SK, NL and US) invited a range of scientists, practitioners and policy makers dealing on a daily basis with biodiversity conservation in terrestrial, freshwater and marine systems to the final workshop. The project leaders generated an excel file with the questions and horizon scanning topics in Hebrew, which was emailed to all of the invitees of the workshop one week in advance for a round of preliminary voting. Each participant scored all of the questions with a 1 or a 0, with 1 indicating the question should be kept and 0 meaning that it should be discarded, having been instructed to choose 50 research questions and 10 horizon scanning topics out of the list. In this preliminary stage, each person voted on all the questions and horizon scanning questions, regardless of the subgroups in which they were placed for the workshop. In the preliminary voting stage, 19 people voted on research questions and 10 people voted on horizon scanning topics.

The workshop leaders assigned a chairperson to each of the 10 topics and sub-groups. Each sub-group chairperson received both the total list of all questions and HS topics prior to the workshop, and individualized lists with the questions and horizon scanning topics, belonging to those topics his/her group would cover in the workshop (two topics per group), arranged in descending order of votes received, in order to review and prepare for the process of elimination and refinement in the first day of the workshop. The preliminary vote results were used for ranking the questions and topics and the ranks were sent to the chairs of each category discussion group with the aim of aiding and increasing the efficiency of the discussion during the workshop.

During the two-day priority questions and horizon scanning topics selection final workshop (May 2011), the 26 invited experts and professionals with a wide range of backgrounds and affiliations participated in the process of selecting the most important research and horizon scanning questions. Participants were invited by the workshop organizers and came from a range of institutions, including governmental organizations, NGOs, civil society, and academics in related fields.

#### The categories for the ten discussion groups

1. Natural and open landscapes—fragmentation, development, construction, corridors; Conservation prioritization.
2. Planning and coordination/liaison including infrastructure, large-scale threats.
3. Nature reserves, threatened populations and species, management, monitoring.
4. Agricultural areas, disturbance, pesticide use, fire and grazing.
5. Policy, governance and regulation, including aquatic and marine systems and marine policy.
6. Marine and aquatic systems, including over exploitation of natural resources—water, gas, pollution etc.
7. Ecosystem services, ecological systems components, economic aspects, ecosystem functioning.
8. Environmental changes: climate change, desertification etc., boundaries and international relations with neighboring countries.
9. Education and increasing awareness, social and cultural aspects, environmental values, human population size, development and building.
10. Invasive species and erupting native species, urban systems.

#### Voting and elimination process at workshop

Participants were pre-divided by the workshop organizers into five discussion sub-groups (4–5 people per group) with the aim of matching individuals to topics close to their areas of expertise and ensuring that each sub-group had a mix of individuals with different organizational affiliations. Each sub-group was assigned two topics. One member of each sub-group was designated as group chairperson prior to the workshop, who received the results of the preliminary voting in advance and coordinated the group discussions and the process of question selection within the group. Another member of each group was assigned the role of secretary to keep minutes during the selection process and to aid in the rewording of questions and horizon scanning topics. During the first day of the workshop, the morning session groups reviewed questions and horizon scanning topics from the group's first topic, and the afternoon session reviewed questions and horizon scanning topics from their second assigned topic. Criteria for selection of questions/horizon scanning topics and workshop instructions, procedures and goals were given in an introductory talk to all participants by the workshop chair (SK).

On the first day of the workshop the participants split up into five small working groups and each group received two categories of questions on which to work. The groups were each given a list of approximately 30 questions on each topic extracted from the total list and were instructed to formulate and choose research questions for which the answer will contribute significantly to biodiversity and nature conservation policy in Israel and that can potentially be answered within a reasonable period of time of a few years. The basic instructions for the horizon scanning section of the workshop included the selection of topics that were important future areas of research and are currently not sufficiently addressed but are likely to become critical to biodiversity conservation policy in Israel in the future. In other words, all groups were instructed to identify future topics and current gaps that may become important in the future (10–25 years ahead) but that are not receiving attention in the policy or research arenas today.

With these guidelines, each group had several hours to review the questions it received for each topic, eliminating some questions, rewording others, combining similar questions, and adding new questions that covered areas not addressed in the original submissions, based on a democratic and inclusive discussion and selection process among all group members. Groups were not committed to selecting or using the questions they received but rather were instructed to use the preliminary list of questions in aiding their top questions’ final phrasing. All groups were instructed to phrase the priority four questions they considered most important for each topic, as well as four runners-up. In practice, groups often chose less than the proposed total of eight priority questions for each topic. During the first day of the workshop, each of the working groups winnowed down the original list of questions and horizon scanning topics through a process of detailed discussion and a democratic vote. While the questions submitted on-line before the workshop were used for discussion in each of the sub-groups, the vast majority of the final questions phrased by each sub-group were different from the original questions submitted, often an integration of several questions, rephrased and/or completely rewritten. The groups also raised and added new questions that were not included in the list of about 30 questions they each received for discussion.

The process of the selection of horizon scanning topics was very similar, with the main difference being that the pool of topics submitted was considerably smaller so there were fewer options to choose from and more room to add additional topics that arose within the groups. The groups were instructed to select one primary horizon scanning topic for each of their two categories, and to determine a single runner-up for each as well. In practice, groups selected 1–3 horizon scanning topics for each of the 10 subjects. Throughout the first day, the workshop coordinator (SK) circulated among the groups, ensuring that two different groups did not have fully overlapping questions, ensured that the process was being followed and aided in the elimination, phrasing and voting processes within groups.

At the end of the first day of the workshop, the original list of 301 research questions had been winnowed down by the workshop participants to 59 rephrased questions. The original list of 35 horizon scanning topics had been narrowed down to 15 rephrased topics. The second day of the workshop consisted of round-table plenary sessions of all participants together in which the final list and wording of the priority research questions was finalized. In the morning session, which constituted the first round of voting, each group chairperson presented the four priority research questions produced by their group during the first day for each of the two topics covered by their group. After each question was presented, a short discussion followed and then all participants voted on whether to retain the question or not; several questions ignited prolonged debate and were moved to the list of runners-up for further discussion later in the day. Following this, each group had time to reword the questions that had been tagged in the plenary discussion as important but in need of changes, including those that had sparked more intense debate during the first round. These reworded and more contentious questions were then re-presented in the next plenary session. Each group chairperson then presented to the plenary for voting the top question (in his/her group’s opinion) from among the runners-up. The top runner-up questions of each group were then voted on. Participants were then given the opportunity to add additional original questions on topics that they felt had not been fully addressed, and these (2–3 in total) were voted on as well.

The total list of all questions that had been voted on, in their updated and altered new wording as phrased in the plenary session, was printed out for all participants with their scores (the percentage of people that had voted to include each question in the final list). Participants were given time to review the list and spot redundancies or problems with wording. Participants then voted on the cutoff point of percentage of people in favor that would qualify a question for inclusion in the final list for Israel. The percentage chosen was 60%, leaving a total of 45 research questions in the final list of priority questions, for each of which at least 60% of participants voted for inclusion (Table 6). The majority of these, however, received much higher percentages of support (>80%); most of the questions were chosen by consensus or by a very large majority. Thus throughout the process of choosing questions, we were flexible about the number of questions we aimed to include in the final list. While the organizers originally aimed for 50 questions, the workshop team decided during the process of question selection in a round table discussion to choose only those questions that received enough votes in a democratic participatory process.

**Table 6:** The top research questions for biodiversity conservation policy in Israel. The list includes the 45 selected questions that were identified as the most important for biodiversity conservation in Israel and received at least 60% of the votes in the final workshop and the topic area/category within which the question was framed. The questions are listed by major thematic areas, as presented in the final workshop outreach phase.

Themes and questions:	
**Threats to biodiversity at multiple spatial scales; Planning, coordination and infrastructure**	1. What are the major threats to biodiversity in Israel at local, regional and national scales, and what is their relative importance and spatial distribution?
	2. What institutional and organizational mechanisms, structures and/or collaborations are necessary for effective conservation of biodiversity in Israel and for ensuring science-based prioritization for legislation, planning and optimal management of open spaces?
	3. How can restoration be effectively implemented in different ecosystems in Israel, and what are the goals and measures of success of such restoration?
	4. How can the effects of intense anthropogenic activity (such as construction, light, noise pollution and quarries) on biodiversity components in Israel and their functioning in built-up and disturbed areas be mitigated or eliminated?
	5. Over time, how does the utilization of different energy sources (such as oil shale, natural gas, solar energy, wind energy and wave energy) affect biodiversity in Israel, and how can negative impacts be minimized?
**Managing and monitoring protected areas, threatened species and populations**	6. What are the impacts of human activities (e.g., tourism and recreational activities) in protected areas on the state of ecosystems and biodiversity and how can the negative impacts of these activities be minimized?
	7. What are the impacts of management activities (e.g., wildfire and clear-cutting, grazing, reintroduction, restoration and non-intervention) and the interactions between them on ecosystems and biodiversity in Israel and how can management be optimized to achieve biodiversity conservation targets?
	8. What are the contributions of existing and planned biosphere reserves and their potential impact on biodiversity conservation in Israel, compared with baseline biodiversity in these areas and other types of protected areas?
	9. What is the relative effectiveness (in economic and professional terms) of different indicators for monitoring Israel's biodiversity and major ecological systems and the changes occurring within them?
	10. What tools and monitoring methods would provide the most effective evaluation of long-term, gradual or incremental changes in the state and functioning of Israel's ecological systems and biodiversity?
**Open landscapes—fragmentation, development, construction, corridors; Conservation prioritization**	11. What are the optimal criteria achievable using scientific tools for determining biodiversity conservation priorities in Israel, including genetic diversity?
	12. How do various land uses in open landscapes (such as agriculture, forestry, and military training grounds) affect biodiversity in Israel, and how can their capacity to maintain biodiversity be improved?
	13. What information about biodiversity is required for effective land use planning, and for determining priorities for biodiversity and nature conservation in Israel?
	14. How should the continuity of open landscapes and ecological corridors be planned to effectively conserve biodiversity and minimize the negative impacts of barriers?
	15. What reciprocal relationships occur between different landscape patches and various land uses at the local scale (e.g., agriculture, forest plantations) that vary in their contribution to local biodiversity conservation, and what is their optimal spatial structure for the conservation of local biodiversity?
	16. What are the biodiversity patterns in Israel at various temporal and spatial scales and what are the predicted future changes in these patterns?
**Agricultural areas, pesticide use, disturbance, grazing and fire**	17. What are the direct and indirect effects of various types of agricultural practices (e.g., conventional, traditional and organic) on biodiversity in Israel?
	18. How do different grazing practices (i.e. grazing pressure and timing) affect biodiversity of various habitats in protected areas and forests in Israel?
	19. How do different management actions influence the resilience and rehabilitation dynamics of biodiversity in different habitats in Israel following fire events?
	20. What are the impacts of various synthetic materials and compounds (such as estrogen analogs, nano-particles, TBT, synthetic nitrogen and pesticides) on biodiversity, and how can these negative impacts be minimized?
**Marine and aquatic systems; Overexploitation of natural resources—water, gas, pollution etc**.	21. What are the impacts of fishing and aquaculture activities on marine and freshwater ecosystems in Israel and what policy responses/actions are needed to address these impacts?
	22. What are the optimal sizes and locations required for marine protected areas (e.g., marine reserves) for effective conservation of Israel’s marine biodiversity and ecosystems?
	23. How can negative impacts of anthropogenic activities on biodiversity be minimized in watersheds and aquatic habitats in Israel and its neighboring countries?
	24. What should be the objectives for the rehabilitation and conservation of aquatic habitats in Israel, and what is the optimal policy for determining the quantity and quality of water that will enable the conservation of aquatic biodiversity?
	25. What are the implications of operating systems, infrastructure facilities (e.g., desalinization plants, power plants, ports, marinas, the Red-Dead Canal) and gas/oil drilling on terrestrial and marine biodiversity in Israel?
**Policy, governance and regulation**	26. What mechanisms will bring about the most effective coordination and cooperation amongst different stakeholders for the purpose of implementing nature and biodiversity conservation policies in Israel?
	27. What measures are necessary to increase transparency and accountability of governance in relation to biodiversity conservation, and what are the current gaps in this area?
	28. In light of the existing legislative and institutional framework, what laws, regulations and enforcement methods are required for the optimal protection of complex ecological systems and local biodiversity in Israel?
	29. What steps should the State of Israel adopt to promote and implement international governance agreements (including multi-sectorial and financial agreements) that will allow efficient management of terrestrial, marine and aquatic ecosystems and will minimize international conflicts?
**Environmental changes: climate change, desertification etc; Boundaries and international relations with neighboring countries**	30. What measures may prove effective in mitigating the negative effects of present and future climate change on distribution ranges and patterns of various biodiversity components across different ecosystems in Israel, and on the interactions amongst these components?
	31. What are the impacts of climate change and anthropogenic activity on ecosystems located in transition zones (ecotones) between the Mediterranean and desert climate-regions of Israel?
	32. How is biodiversity in Israel affected by the state of nature and anthropogenic activity (e.g., hunting, agriculture) in neighboring countries?
**Ecosystem services: ecosystem components, ecosystem functions and economic aspects**	33. How are the various components of biodiversity involved in supplying and shaping ecosystem services in Israel, including agricultural and urban systems?
	34. What is the relationship between human welfare and the services provided by different ecosystems in Israel?
	35. What economic tools, such as cost-benefit analyses of non-market ecosystem services, will effectively encourage biodiversity conservation in Israel?
	36. How can sustainable development be implemented in order to maximize native biodiversity and the provision of ecosystem services at different spatial and temporal scales?
	37. What is the role of soil biodiversity in providing terrestrial ecosystem services in Israel?
**Invasive and expanding species, urban systems**	38. Which tools are most effective in preventing the entry and establishment of potential invasive species in terrestrial and marine habitats in Israel?
	39. Which invasive and expanding species have the most significant negative impact on biodiversity in Israel and what are the most effective tools for dealing with these species?
	40. What are the positive and negative impacts of urban nature on biodiversity in Israel, and how can urban areas be managed to increase native biodiversity?
**Education and awareness, social and cultural aspects, values, human population size and consumption**	41. How knowledgeable is the Israeli public about biodiversity, and what are its values and attitudes to this topic?
	42. How can a change of perception of the importance of biodiversity and nature conservation be achieved among the general public, policy makers and the media in Israel?
	43. What are the effects of human population growth and consumption (including goods, construction and transportation) on biodiversity and nature conservation in Israel, and what are the policy alternatives for minimizing their impacts on biodiversity in Israel?
	44. How can interactions between scientists and the public, practitioners and policy makers in the field of biodiversity and nature conservation in Israel be strengthened?
	45. How can stakeholders and decision makers be mobilized towards actions that will increase knowledge and awareness of biodiversity and the state of biodiversity in Israel?

At the end of the workshop the participants held a discussion on the lessons learned from the process undergone in the workshop and potential following steps, outreach plans, the practical application and funding for the research questions and ways to apply the results of this theoretical exercise into policy-making and in shaping research priorities and future projects. One of the main topics discussed at the end of the workshop was the identification of the community for which the outreach of the workshop outcomes should be intended, including academia, government ministries, funding agencies, managers and others. The project leaders took upon themselves to lead the continuing activity of making the products of the workshop accessible to these target groups.

While the horizon scanning topics were included in the sub-group discussions and were presented shortly in the plenary session, due to shortage of time, the final vote on the horizon scanning topics was completed via email in the week following the workshop voting and discussions among all the workshop participants, using the same threshold of 60% of the votes for a horizon scanning topic to be included in the final list. Eight final horizon scanning topics were selected in five major categories (see Table 7). The 45 top research questions and eight horizon scanning topics (in Hebrew) [20] were then professionally language-edited and were translated to English by the project leaders and an English-language professional editor.

**Table 7:** The top Horizon Scanning topics for biodiversity conservation policy in Israel. The final eight horizon scanning topics that were identified as the most important for biodiversity conservation in Israel. The topics are listed by major categories.

Themes and topics:	
**Climate change**	1. Prediction of the impacts of the most extreme IPCC scenarios (such as maximum increase in average global temperature, maximum sea level rise and extreme drying out of ecosystems) on biodiversity in Israel
	2. Adaptation, planning and management of the protected area system in Israel (including nature reserves, national parks and forests) in accordance with predicted future climate changes.
**New threats to terrestrial biodiversity**	3. Prediction and mapping of future threats to biodiversity in Israel resulting from population growth and increased consumption, including growing use of renewable energy sources (e.g., wind turbines and solar farms) and new types of transportation (e.g., electric cars and personal aircraft).
	4. Development and application of advanced and sophisticated technologies for locating and dealing with invasive and expanding species
**New threats to marine and aquatic biodiversity**	5. The study, monitoring and evaluation of new ecosystems created around human-made infrastructures in marine and coastal areas and their impact on species composition and ecosystem functioning; preparation for minimizing the negative impacts of these new infrastructures on biodiversity in Israel.
	6. Establishment of a legal and institutional framework for marine and aquatic biodiversity conservation under threat (e.g., gas extraction and artificial islands).
**Agricultural changes**	7. Predicting future changes in agriculture (e.g., abandonment of agricultural lands, transition to intensive agricultural practices in other areas, and the expansion of covered/greenhouse agricultural areas), the expected impact of these changes on biodiversity and their consequences for open landscape planning policies.
**Geopolitical changes**	8. Understanding the effects of geopolitical changes in Israel and amongst its neighbors (e.g., future peace agreements, evacuation of settlements) on biodiversity in Israel.

#### Generation and dissemination of products (outreach)

The final list of questions was edited by the leading team of the project (SK, NL and US) and was produced as a brochure that was uploaded on the Internet in both Hebrew and English. The project leaders processed the results, divided them into final thematic areas (partly corresponding with the topics of the discussion sub-groups of the workshop; see Tables 6 and 7) and worked on outreach of the outcomes to the relevant organizations in Israel. The final phase of the project, which we consider perhaps the most important one, included wide dissemination of the selected questions and horizon scanning topics among scientists, practitioners, managers and policy makers. The workshop organizers wrote a short executive summary and, with the help of a professional graphics designer, published on the projects web page a report (in both Hebrew and English) aimed at decision and policy makers, as well as practitioners in Israel based on the results and products of the workshop. We distributed the results in Israel among the organizations involved in the exercise and dozens of additional policy makers, scientists, Chief Scientists of several government offices (e.g., Ministry of Environmental Protection, Ministry of Energy and Water), planners, managers and the general public via email, mailing lists, handouts at conferences, personal meetings, seminars etc. With the help of a professional environmental policy consultant, we held a range of meetings, seminars and talks with relevant governmental agencies, nongovernmental and academic organizations to discuss the results of the project and their application. We linked the outcomes to broader policy making (see also Sutherland and Woodroof 2009).

## Results

### General patterns across all projects

The number of questions selected in the different projects compared here ranged from 40 to 100 (Table 1) and the number of selected horizon scanning topics ranged from 8 to 25 (Table 2). The two earliest projects completed (including the UK [3] and global questions [6]) both identified a larger number of priority questions (100) than the more recent projects (40–45 questions for the US [4], Canada [5], Switzerland [11] and Israel (this paper)). In the latter, forty-five questions and eight horizon scanning topics (divided into five broader categories) were identified as the most important for biodiversity conservation policy in Israel [20]. These priority issues/questions received at least 60% of the participants’ votes at the final workshop of the project (see Methods) and are presented in Table 6.

Most projects included here focused on issues related to conservation and management of biodiversity. When comparing the outcomes of the various projects and those of the questions vs. horizon scanning projects by categories, several patterns emerge. In general, the percentage of priority topics selected per category had strong correlations across regions for both the questions and for the horizon scanning projects examined here (Tables 3 and 4). For the priority question projects, we found that the US’s questions (in 2011 as well as in 2014) were the least correlated to other questions projects undertaken around the world (Table 3). This mainly resulted from the fact that the US project had relatively few priority questions in the proactive class. The correlations amongst the different horizon scanning exercises did not show clear differences (Table 4). When comparing the distribution of the selected topics of the different categories of the priority question versus the horizon scanning projects (for regions in which both were conducted), we find intermediate correlations for both Israel (0.71) and the UK (0.51 and 0.69), whilst the correlations between the global questions and horizon scanning projects were 0.46 for the Global 2010 horizon scan, and 0.28 for the Global 2011 horizon scan.

### Thematic results

We detail below the results for those topic categories (see Materials and Methods) in which clear differences between projects were found.

#### Climate change

The percentage of questions referring to climate change ranged from 4.4% to 30% of the total top questions selected in the projects completed in different countries. In comparison, the total percentage of horizon scanning topics dealing with climate change ranged from 25% to 46.7% of all topics identified per project. In most of the projects examined here, climate change received a stronger emphasis in horizon scanning compared with questions projects (Tables 1 and 2). Similarly, when comparing results of top questions and horizon scanning projects completed in one region (i.e. for the global, UK and Israel projects), climate change was always more important in the top horizon scanning topics compared with the priority questions identified (Table 5). In the UK, the percentage of questions dealing with climate change was almost double that for the priority policy options for UK nature conservation questions published in 2010 (20.3%) compared with the top policy-relevant ecological questions project published four years earlier (11%, see Table 1).

The strongest emphasis on climate change was found in the US 2014 conservation science and policy project (30% of all priority questions dealt with climate change; see Table 1). In Israel, only 4.4% of the total top 45 questions selected dealt with this topic. However, a significantly larger portion of the selected horizon scanning topics in Israel (25%) dealt with climate change, with an Odds Ratio of 7.24 comparing the proportion relating to climate change in the questions vs. horizon scanning for Israel.

#### Socio-political issues

Overall, socio-political issues were explicitly included in about a quarter of the top questions identified in most projects (with the highest value being 40% for Canada, Table 1). The only project that was substantially lower was the UK ecological questions (2006), in which just 6% of the questions included socio-economic issues. The proportion of questions that included human-related systems was relatively high in all cases and ranged from 15% in Canada to 52.5% in the 2014 US project. However, a relatively small proportion of the questions in most projects referred to human demography, ranging from nil to 7.5%. In the horizon scanning projects, socio- political issues received higher attention in the UK (40%) and Israel (37.5%) compared to some of the global scale projects (6.7% in 2010 and in 2012, and 0% in 2013). Human related systems and human demography both received relatively high attention in horizon scanning projects, equal or higher than 40% in all global projects and 62.5% (Israel) of the top selected horizon scanning topics dealing with aspects of human related systems.

#### Marine environments

Overall, marine issues were more common in the selected horizon scanning topics compared with the top questions (Table 5). The percentage of questions dealing with marine systems was especially high in the horizon scanning projects of Israel and the 2010 and 2012 global horizon scanning project, where 37.5%, 53.3% and 40% of the horizon scanning topics, respectively, referred to marine systems. In both projects it had a higher percentage in horizon scanning than in the top questions. In both Israel and in the global (2009) question projects, 11% of the questions referred to marine systems. The Odds Ratio comparing the results of the proportion of questions in the marine category in the Israel horizon scanning versus questions project was 4.85. The proportion of questions dealing with marine issues was lower for Canada, with only three of the final top 40 questions (7.5%) referring to marine issues (Table 1), as well as in the UK’s 2010 conservation policy questions, where only 7.2% (5 of 69) of the questions dealt with marine issues. In Switzerland, a land-locked country, no questions referred to marine issues. When comparing the percentage in the global horizon scanning projects (2010, 2011) with the results of the global questions project, in both cases the percentage of topics dealing with marine systems was higher for the horizon scanning project compared with the priority questions project, and especially when comparing the 2010 horizon scanning projects with the results of the 2009 global questions project (Table 5).

#### Freshwater systems

The percentage of questions addressing freshwater issues in the priority questions projects ranged from 7.5% in the US questions project to 26.1% in the UK 2010 questions project (Table 1). Most of the Global horizon scanning projects had the highest proportion of questions that dealt with freshwater systems (26.7%, Table 2), compared with all the projects. Just 12.5% of the top questions referred to freshwater issues in Israel (see Table 1).

#### Ecosystems services

Ecosystem services received much higher emphasis in the questions projects compared with horizon scanning projects when comparing the 2010 UK questions and horizon scanning projects (Table 5). Ecosystem services received especially high emphasis in the global (2009) questions (18%), followed by the Canadian (2010) questions project (17.5%), and by the UK conservation policy (2010) questions project (17.4%). In Israel, ecosystem services-related issues received relatively limited attention until recently, as reflected by their being included in 11% of the selected questions (5 of 45 questions), and no horizon scanning topics addressed ecosystem services in Israel (Table 2). However, since the project was completed, several initiatives aimed at increasing awareness of the importance and potential of incorporating ecosystem services considerations into research and conservation decisions have been initiated in Israel, which are currently undergoing and will likely increase the awareness of this topic (see Discussion).

#### Cross-boundary issues

The proportion of selected questions and horizon scanning topics referring to cross-boundary issues and neighboring country related issues was higher in Israel (8.9%) than in any other regions and projects, closely followed by the 2009 Global questions (8%) and the US 2011 questions project, in which cross-boundary issues were included in 7.5% of the top 40 questions (Table 1). Cross-boundary issues were absent from the UK's and Switzerland’s (0%) and very low in Canada’s (2.5%) selected ecological questions and horizon scanning projects (Table 2).

#### Proactive questions and topics

The number and percentage of proactive questions and topics was relatively high in most projects, with 12.5%-75.6% of the selected questions or horizon scanning topics being proactive. The relative proportion of proactive issues was lower in the horizon scanning compared to the questions exercises (Table 5). The 2011 global horizon scanning project [25] was an exception, with only two of the 15 topics being proactive, although the global project published a year earlier [26] had far higher percentages (40%; 6 of 15 topics, see Table 2). The US’s selected questions had the lowest percentage of proactive questions (22%) compared to all other questions projects, which had a much higher representation of proactive issues in the top questions (45%-75.6%). The most proactive approach was found for Israel, where 75.6% of all questions and 50% of all horizon scanning topics had a proactive component (Tables 1 and 2).

## Discussion

In the past decade, several projects aiming at identifying priority questions and/or horizon scanning topics have been completed across multiple countries and regions (e.g., [3–11]). However each paper has been presented in isolation. This is the first attempt to bring these studies together in a more synthetic manner. This paper presents a comparison of the projects completed in recent years (between 2006–2014), addressing both questions and horizon scanning project outcomes. The emphasis of the questions and issues selected depends on a wide range of factors. These include, for example, the specific biodiversity and ecosystem under consideration, the threats to biodiversity, the conservation priorities in each region, the social, economic, historical, cultural and political context, the specific composition of the participants who selected the priority questions or horizon scanning topics and many other factors that are difficult to quantitatively separate. For example, policy and management oriented participants may well emphasize socio-political issues compared to ecologists and other natural scientists. While it is difficult to compare the outcomes of the projects quantitatively and to tease out the exact factors shaping the differences found, better mapping and understanding of the variation among projects can be informative and useful for planning and execution of future related projects worldwide. While each project defined its own specific goals and methodology, many of them share similarities in the final methods used. As an example, in all projects practitioners and policy makers took part in a final workshop to identify the final list of questions or topics.

While the projects aiming to identify the top questions are relatively similar to each other in framework and goals, horizon scanning is often less straightforward; it is less intuitive and more challenging, with its definitions and goals being more diverse. We propose that future projects present the methodology used in a more explicit and repeatable way (e.g., adding the exact question solicitation details, the composition of the question selection team, how the questions were coded etc.) to allow comparison between the results of different projects over both space and time. We propose that it is as important to describe the precise methods for such exercises as for any sampling or experimental procedure that would normally be described in the methods in sufficient detail to allow it to be repeated.

A key issue is whether such exercises provide a synthesis of the current state of knowledge in a repeatable manner or whether these are simply the consequence of the individuals who happen to be in the room during the discussion. The process is designed in order to minimize the impact of single individuals, for example by having an extensive team of experts, by having pre-workshop voting and by having a systematic iterative process of discussion and voting. The fact that there are similar patterns in the different projects, and that the variability can be explained by differences in the requirements of different regions, helps confirm the validity of the approach.

The project presented here for Israel was the first to undertake priority questions and horizon scanning exercises simultaneously, using the same methods, coordinators, and participants in a single framework. This has several advantages, such as a more reliable comparison of the outcomes from the two projects, and allowing the projects to be complementary to each other. Indeed, it was easier to delineate the difference between current questions and horizon scanning topics in Israel compared with other regions in which both exercise took place yet were done by different teams at different times (e.g., globally and in the UK). Thus, having the two projects proceed simultaneously has the advantage of better differentiating between current and future priorities and thus assigning the questions to the appropriate project. However, it did make the project and process more complex and likely led to less time being allocated to the horizon scanning project in Israel, which was more challenging for participants. It is often more challenging for experts to identify issues that will be important in the future than issues that are already more clearly apparent and are crucial to address immediately. This is especially true in the context of biodiversity conservation, where there are high levels of complexity and uncertainty. For example, the definitions of horizon scanning, the number of topics identified and the time frame set for identification of key horizon scanning issues differ between different horizon scanning projects completed in recent years (see variation in definitions for horizon scanning in S2 Appendix). This likely impacted some of the differences in outcomes of different projects undertaken.

In this paper, we found that when comparing priority questions to horizon scanning topics, there are clear differences among exercises in the emphasis given to some issues, most notably to climate change and marine ecosystems. In Israel (and to a lesser degree in other exercises), climate change-related issues received relatively limited attention in the priority questions project, where the questions identified referred to current important issues that if addressed, will have the highest impact on biodiversity conservation. However, climate change received significantly more attention when identifying horizon scanning topics which are likely to be important in the future. It is interesting to find that climate change is still perceived as a future challenge rather than a current important issue to address in Israel (see below). This illustrates how the precise framing of the exercise influences the outcome.

Similarly, in all cases, marine issues received more attention in the selected horizon scanning topics (future issues to address) than in the current top questions. Several ongoing projects are currently specifically focusing on the identification of priority questions for marine environments (e.g., globally and in Canada [9]). In the horizon scanning projects of Israel and the 2011 global horizon scanning project [25], the percentage of selected questions dealing with marine issues was relatively low (Table 2). This may reflect the fact that marine systems are still receiving less attention in current conservation prioritization, yet awareness of the importance of marine environments is growing with the increasing threats to marine biodiversity [27, 28] and the clear need to better prioritize conservation efforts and policy in marine, in addition to terrestrial systems. For example, in Israel, there have been recent discoveries of natural gas in the Mediterranean Sea in the economic waters (EEZ) of Israel, a natural resource that is expected to become the major source of the country's energy in coming decades [29]. This has led to a surge of recent public, media, economic and political interest in planning the sea and coastal systems, and in the environmental threats caused by drilling the gas and transporting it to the coast. In addition, the water crisis, following a series of drought years in Israel, has shifted attention and funding to the construction and operation of desalinization plants in the Mediterranean Sea, which has become a major source of freshwater for the country in recent years and is planned to increase in coming years [30,31]. Interestingly, there was an increase in the attention given to marine issues in the 2011 global horizon scan [25] compared with that in 2010 [26]. This may be related to increased attention to deep ocean conservation issues. The difference may also be partly related to the relatively small number of topics in total (15) [25,26].

While marine issues received relatively limited attention in the UK 2010 top questions project, with only 5 of 69 questions (7.2%) dealing with marine issues, relatively high emphasis was given in the UK in 2010 to freshwater issues with 18 questions (26.1%) dealing with these related issues. In the UK, the intensity of land use, and periods of water shortage, with subsequent impacts on aquatic systems, combined with the high and increasing awareness of the importance of the diversity of wetland-type ecosystems and the threats posed to these environments by global changes [32] may have led to relatively high awareness of these issues in more recent projects.

We found large variation in the emphasis given to climate change issues amongst different projects. In the US over a quarter of the top 40 questions were related to climate change issues, while in the project completed in Israel, only two of the total 40 top questions (4.4%) dealt in any way with climate change. It is hard to determine the factors that led to this result, but it may reflect the fact that Israel is lagging behind Europe and the US in the attention directed in the past decade to climate change in the context of conservation in both the scientific, policy and management communities. Due to the high inter-annual variability of rainfall in Israel, the expected variation in rainfall resulting from global warming has been difficult to predict [33], though some clear temperature-related trends are slowly accumulating [34,35]. Nevertheless, as predicted, the percentage of horizon scanning topics dealing with climate change in Israel greatly exceeded that of the questions. This may reflect increasing interest in the topic in recent years, as reflected by the recent establishment of a new climate change research center in Israel funded by the Ministry of Environmental Protection, and Israel presenting its Second National Communication on Climate Change in 2010 under the United Nations Framework Convention on Climate Change [15]. This is an interesting area that deserves further interdisciplinary research.

In recent years, there has been increasing attention towards the importance of incorporating cross-boundary issues into conservation and understanding the effect of collaboration on conservation outcomes [36,37]. These topics are especially relevant in areas where countries share borders and where collaboration across these borders is challenging (e.g., due to political issues). Indeed, as predicted, we found that the highest emphasis to cross-boundary issues was given in Israel compared to most other projects in both the questions and horizon scanning topics. Israel is a small and narrow country with long borders that deals daily with issues related to its neighbors in regards to environmental and policy topics, so increasing awareness of the importance of collaboration and cross boundary impacts on its biodiversity and conservation planning. Dealing with challenges such as limited freshwater, invasive species, and conservation of threatened species directly depends on how these issues are addressed in neighboring countries across the region (e.g., [38]). Surprisingly, cross boundary issues were entirely absent from the Swiss (2012) project. Cross-boundary issues were mostly absent also from the UK's selected ecological questions and horizon scanning topics and none of the 69 questions in the UK conservation policy project (2010) were related to cross-boundary issues; probably due to the only land boundary being with the Republic of Ireland.

Ecosystem services issues were emphasized more in the questions projects than in the horizon scanning projects (Table 5), possibly because the general problem has been identified, but the specifics and means of delivering ecosystem services often remain unclear. When comparing the original UK project ([3]; ecological questions) with that published for the UK in 2010 (conservation policy), we find a substantial increase in the attention given to ecosystem services related issues (from 4% to 17.4%). This may be due to the fact that in the UK ecological questions project took place earlier than the others, in 2005, and interest in this subject has grown substantially in recent years (e.g., [39]). Nevertheless, in most cases ecosystem services are not yet incorporated in depth into conservation assessments [40]. Contrary to the pattern seen for climate change and marine systems, when comparing the outcomes of the horizon scanning with the current questions, it is interesting to see that more emphasis is given to ecosystem services in the priority questions compared with horizon scanning topics (for example in Israel and in the global projects). This may reflect a notion that this subject is already being dealt with and therefore does not represent a major gap for the future. The difference between the proportions of selected questions in Israel and the recent UK projects with regard to ecosystem services may be related to the fact that the UK has commissioned a National Ecosystem Assessment (http://uknea.unep-wcmc.org/), whereas in Israel, the issue of ecosystem services is still at very initial stages amongst scientists, policy makers and practitioners circles, and the Israeli National Ecosystem Assessment is still under preparation, with one of its main aims being to raise awareness to the benefits humans gain from ecosystem services.

Interestingly, although human population growth and demographic changes are often a major threat to biodiversity and ecosystems, human demography received relatively limited attention in all projects, with the percentage of questions dealing directly with demographic issues ranging from zero (for both the two UK and the Swiss priority questions projects) to just 5–7.5% (in the US, Canada and global question projects). While demography and population increase is a major issue in Israel’s conservation challenges, where human population size has increased in the past 60 years tenfold and population growth rates are some of the highest in the world [41], only one (2.2%) of the priority questions selected in Israel directly dealt with this issue. This may result from the reluctance of many scientists, managers and policy makers to include socio-politically sensitive and religion/cultural-related issues [41,42], preferring to address them in an indirect way (see discussion in the special *Science* 29 July 2011 issue on the topic “Population”). Indeed, human demography received relatively more attention in the horizon scanning projects, suggesting this is possibly viewed as a topic that should be addressed in the future in more detail. We propose that future projects try to address and incorporate these important factors more explicitly. One possible way to enhance this topic could be by making an effort at identifying and inviting to the project workshops leading experts in these areas, scientists, practitioners and policy makers, who also have some experience in environmental decisions, and ideally in conservation-related issues.

Future projects may benefit from relating the questions and horizon scanning exercises and their outcomes to the Convention for Biodiversity (CBD) and the Aichi biodiversity goals adopted by the CBD parties in 2010 (UNEP, 2010). Aichi goals and targets are used as a guidance tool for various governments and ministries in forming national biodiversity strategic and action plans, as well as National Biodiversity Strategies and Action Plans (NBSAP; https://www.cbd.int/nbsap). While performing priority questions or horizon scanning projects in the future, teams can choose to build the categories of the questions or analyze the questions in such manner that they can relate to existing global policy and cover also some of the underlying drivers of biodiversity loss. Relating the questions to international and local goals and targets in NBSAPs can also be used as a tool for communicating scientific issues to policy makers and practitioners in order to help mainstream the outcome of the project. This can help enhance the connections between scientific expertise and ongoing policy processes taking place globally and regionally.

In general, we found that most projects completed to date had relatively high ratios of proactive questions, with the exception of the US project [4]. This was especially true for Israel, where the number and percentage of proactive questions was very high. Over 70% of the questions selected in Israel included a proactive component (Table 1). The proportion was also relatively high for Israel's top horizon scanning topic, with 50% of the topics having a proactive component. This likely results from the fact that Israel's biodiversity is facing major threats due to the increasing human population size [43] and a clear concern of the participants that this exercise identified means of producing solutions. This may also partly result from the composition of the working group that determined the questions, which consisted of several “hard-core” practitioners from conservation NGOs. The higher proportion of proactive priority questions compared to the proportion in the horizon scanning projects likely results from differences in the aims of the projects. It may be easier to provide proactive phrasing of questions, where one already knows something about how they can be solved, compared with the less clear and often unknown future open challenges defined in the horizon scanning exercises.

In coming years, additional countries and regions will perform projects aiming at identifying priority questions and/or horizon scanning topics for conservation and other environmental issues. Indeed, this methodology is increasingly being applied in a wide range of other fields and this seems likely to increase. This paper provides one of the first overviews and comparisons of the multiple top questions and horizon scanning projects completed to date. It is important to note that the different projects were not all done at the same time and are not independent. More recent projects have often used the experience and knowledge gained from previous ones, and some participants took part in more than one project. The teams in each project were largely composed of different people, coming from different organizations, and had a different proportion of scientists, practitioners, policy makers and other stakeholders. We suggest that their comparison should mostly be used to provide insights to the process rather than to draw fine conclusions as to differences between countries and projects. Future projects will benefit from allowing simultaneous identification of both questions and horizon scanning topics, as done here for Israel. In addition, future projects can make more use of current communication technologies (e.g., Facebook, Twitter), to enhance the breadth of participants in the initial question-soliciting phases of the project. We propose that future projects examine the outcomes and outreach phases of the projects already completed globally and incorporate lessons from these into their project planning and execution (e.g., [44]). This may enable better application of the outcomes of the projects, further enhancing the ability to confront current and future threats and challenges in conservation and bringing together scientists, policy makers and practitioners. We also propose that future horizon scanning projects define their targets, aims and the future time frame they refer to very clearly to help participants creatively think years ahead, while gaining insight from already completed projects. We hope that future projects explicitly include practical factors into the question phrasing, such as quantitative assessments of the scale, feasibility, resources and time required to address each of the questions. This could provide guidance to policy makers and managers as to how to best use the outcomes of the questions and horizon scanning projects in future work, research planning, prioritization, management and policy.

In addition to providing the first such comparison of outcomes across multiple projects, this paper presents the first project that included a Mediterranean country (Israel). While it is too early to tell what impact the project will have on science-based conservation and policy in Israel, the process itself, in which hundreds of people took part, already had a significant impact in that it required a large number of participants from a wide range of organizations to both individually and collaboratively address the topic and explicitly come up with important questions and future issues and to prioritize among them. We expect that, along with other ongoing and completed projects globally, this will constitute a significant contribution to attempts to clarify and prioritize the most important questions and horizon scanning topics to address for better conservation of biodiversity.

## Summary and Main Conclusions

- This project provides the first synthesis of the outcomes of the different priority questions and horizon scanning projects published in recent years (2006–2014).
- While projects show relatively strong correlations in their outcomes, the number of top questions selected ranges widely between 40 and 100 and some factors show major differences in the emphasis they receive.
- The global top questions exercise outcomes show the highest correlation with the outcomes of other exercises with the exception of the project completed in the US.
- Climate change, human demography and marine issues were more important in the horizon scanning topics compared with the priority questions identified when compared for the same region, suggesting these topics are still perceived as future threats more than current issues to address.
- Ecosystem services show the opposite pattern by being more emphasized in the questions projects compared with the horizon scanning projects.
- Cross boundary issues are more emphasized in Israel and the US than in other regions that have fewer neighboring countries (e.g., the UK).
- A strongly proactive approach is seen in Israel, especially as compared with the US project outcomes.
- Future questions and horizon scanning projects should aim to explicitly emphasize interdisciplinary approaches, such as socio-economic ecological issues, being key components in determining conservation biodiversity priorities.
- Future projects should aim to include priority questions and horizon scanning in one framework to allow comparison, explicitly and clearly defining the goals and expected outcomes of horizon scanning efforts.
- Given that these projects often involve policy makers and managers and may have actual impact on conservation actions, we propose that they can benefit from previous experience and save time and resources by adopting the lessons from previous such projects completed around the world.

## Supporting Information

S1 Appendix A. List of organizations in Israel from which research questions and horizon scanning topics were submitted. B. List of organizations that participated in the priority questions and horizon scanning topic selection workshop in Israel.
(DOCX)

S2 Appendix The definitions of horizon scanning (HS) in each of the horizon scanning in the projects included in this study. The definition, while sharing some attributes, differed among the projects conducted globally and compared here, which may have partly affected their different outcomes.
(DOCX)

## References

[1] Sutherland WJ, Freckleton RP (2012) Making predictive ecology more relevant to policy makers and practitioners. Philos Trans R Soc Lond B Biol Sci 19: 322–330. 10.1098/rstb.2011.0181 PMC3223805 22144394

[2] Rudd MA (2011) How research-prioritization exercises affect conservation policy. Conserv Biol 25: 860–866. 10.1111/j.1523-1739.2011.01712.x 21790784

[3] Sutherland WJ, Armstrong-Brown S, Armsworth PR, Brereton T, Brickland J, Campbell CD, et al (2006) The identification of 100 ecological questions of high policy relevance in the UK. J Appl Ecol 43: 617–627.

[4] Fleishman E, Blockstein DE, Hall JA, Mascia MB, Rudd MA, Scott JM, et al (2011) Top 40 priorities for science to inform US conservation and management policy. Bioscience 61: 290–300.

[5] Rudd MA, Beazley KF, Cooke SJ, Fleishman E, Lane DE, Mascia MB, et al (2011) Generation of priority research questions to inform conservation policy and management at a national level. Conserv Biol 25: 476–484. 10.1111/j.1523-1739.2010.01625.x 21175828 PMC3108069

[6] Sutherland WJ, Adams WM, Aronson RB, Aveling R, Blackburn TM, Broad S, et al (2009) One hundred questions of importance to the conservation of global biological diversity. Conserv Biol 23: 557–567. 10.1111/j.1523-1739.2009.01212.x 19438873

[7] Pretty JJ, Sutherland WJ, Ashby J, Auburn J, Baulcombe D, Bell M, et al (2010) The top 100 questions for global agriculture and food. Int J Agr Sustain 8: 219–236.

[8] Ingram JSI, Wright HL, Foster L, Aldred T, Barling D, Benton TG, et al (2013) Priority research questions for the UK food system. Food Security 5: 617–636.

[9] Fissel D, Babin M, Backmayer R, Denman K, Dewailly E, Gillis KM, et al (2012) 40 priority research questions for ocean science in Canada: A priority-setting exercise by the core group on ocean science in Canada. The Council of Canadian Academies, Ottawa ON.

[10] Sutherland WJ, Freckleton RP, Godfray HCJ, Beissinger SR, Benton T, Cameron DD, et al (2013) Identification of 100 fundamental ecological questions. J Ecol 101: 58–67.

[11] Walzer C, Kowalczyk C, Alexander JM, Baur B, Bogliani G, Brun J-J, et al (2013) The 50 most important questions relating to the maintenance and restoration of an ecological continuum in the European Alps. PLoS ONE 8(1): e53139 10.1371/journal.pone.0053139 23341928 PMC3544812

[12] Sutherland WJ, Bailey MJ, Bainbridge IP, Brereton T, Dick JTA, Drewitt J, et al (2008) Future novel threats and opportunities facing UK biodiversity identified by horizon scanning. J Appl Ecol 45: 821–833.

[13] Sutherland WJ, Alves JA, Amano T, Chang CH, Davidson NC, Finlayson CM, et al (2012) A horizon scanning assessment of current and potential future threats facing migratory shorebirds. Ibis 154: 663–679.

[14] Sutherland WJ, Allison H, Aveling R, Bainbridge IP, Bennun L, Bullock DJ, et al (2012) Enhancing the value of horizon scanning through collaborative review. Oryx 46: 368–374.

[15] Axelrod MY (2010) Israel's Second National Communication on Climate Change—Submitted under the United Nations Framework Convention on Climate Change. Jerusalem: The Israel Ministry of Environmental Protection.

[16] Levin N, Lahav H, Ramon U, Heller A, Nizry G, Tsoar A, et al (2007) Landscape continuity analysis: A new approach to conservation planning in Israel. Landsc Urban Plan 79: 53–64.

[17] Naveh Z, Dan J (1973) The human degradation of Mediterranean landscapes in Israel In: di Castri F, Mooney HA, editors. Mediterranean Type Ecosystems. Heidelberg: Springer pp.7, 373–390.

[18] Zohary D, Hopf M, Weiss E, (2001) Domestication of Plants in the Old World. Oxford: Oxford University Press.

[19] Safriel UN (2010) Israel’s National Biodiversity Plan. Jerusalem: Israel Ministry of Environmental Protection.

[20] Kark S, Levin N, Shanas U (2012) The most important research questions for biodiversity conservation policy and horizon scanning (in Hebrew with English abstract). Ecology and Environment 3: 132–133.

[21] Potchter O, Saaroni H (1998) Examining the climatological classification of Israel according to Koeppen’s classification. Studies in the Geography of Eretz-Yisrael. 15:179–194 (in Hebrew).

[22] Safriel UN (2006) Dryland development, desertification and security in the Mediterranean In: Kepner WG, Rubio JL, Mouat DA, Pedrazzini F, editors. Desertification in the Mediterranean Region: a Security Issue. The Netherlands: Springer pp. 227–250.

[23] Rita H, Komonen A (2008) Odds ratio: an ecological sound tool to compare proportions. Annales Zoologici Fennici 45: 66–72.

[24] Abramson J, Abramson ZH (2011) Research Methods in Community Medicine: Surveys, Epidemiological Research, Programme Evaluation, Clinical Trials. 6th Edition John Wiley and Sons ISBN: 978-0-470-98661-5.

[25] Sutherland WJ, Bardsley S, Bennun L, Clout M, Côté IM, Depledge MH, et al (2011) Horizon scan of global conservation issues for 2011. Trends Ecol Evol 26: 10–16. 10.1016/j.tree.2010.11.002 21126797

[26] Sutherland WJ, Clout M, Cote IM, Daszak P, Depledge MH, Fellman L, et al (2010) A horizon scan of global conservation issues for 2010. Trends Ecol Evol 25: 1–7. 10.1016/j.tree.2009.10.003 19939492

[27] Pereira HM, Leadley PW, Proenca V, Alkemade R, Scharlemann JPW, Fernandez-Manjarrés JF, et al (2010) Scenarios for Global Biodiversity in the 21st Century. Science 330: 1496–1501. 10.1126/science.1196624 20978282

[28] Kark S, Brokovich E, Mazor T, Levin N (2015). Emerging conservation challenges and prospects in an era of offshore hydrocarbon exploration and exploitation. *Conservation* Biology 29: 1573–1585. 26219342 10.1111/cobi.12562

[29] Shaffer B (2011) Israel—New natural gas producer in the Mediterranean. Energy Policy 39: 5379–5387.

[30] Tal A (2011) The desalination debate—lessons learned thus far. Environment: Science and Policy for Sustainable Development 53: 34–48.

[31] Feitelson E, Rosenthal D (2012) Desalination, space and power: The ramifications of Israel’s changing water geography. Geoforum 43: 272–284.

[32] Clarke SJ (2009) Adapting to Climate Change: Implications for Freshwater Biodiversity and Management in the UK. Freshw Rev 2: 51–64.

[33] Morin E (2011) To know what we cannot know: Global mapping of minimal detectable absolute trends in annual precipitation. Water Resour Res 47: W07505.

[34] Samuels R, Smiatek G, Krichak S, Kunstmann H, Alpert P (2011) Extreme value indicators in highly resolved climate change simulations for the Jordan River area. J Geophys Res Atmos. 116: D24123.

[35] Shohami D, Dayan U, Morin E (2011) Warming and drying of the eastern Mediterranean: Additional evidence from trend analysis. J Geophys Res Atmos 116: D22101.

[36] Kark S, Levin N, Grantham H, Possingham HP (2009) Between-country collaboration and consideration of costs increase conservation planning efficiency in the Mediterranean Basin. Proc Natl Acad Sci U S A 106: 15360–15365. 10.1073/pnas.0901001106 PMC2741257 19717457

[37] Mazor T, Possingham HP, Kark S (2013) Collaboration among countries in marine conservation can achieve substantial efficiencies. Divers Distrib 19:1380–93.

[38] Shanas U, Abu Galyun Y, Alshamlih M, Cnaani J, Guscio D, Khoury F, et al (2006) Reptile diversity and rodent community structure across a political border. Biol Conserv 132: 292–299.

[39] Potschin MB, Haines-Young RH (2011) Ecosystem services: Exploring a geographical perspective. Prog Phys Geogr 35: 575–594.

[40] Egoh B, Rouget M, Reyers B, Knight AT, Cowling RM, van Jaarsveld AS, et al (2007) Integrating ecosystem services into conservation assessments: A review. Ecol Econ 63: 714–721.

[41] Orenstein DE, Hamburg SP (2009) To populate or preserve? Competing political-demographic and environmental paradigms in Israeli land use planning. Land Use Policy 26: 984–1000.

[42] Orenstein DE (2004) Population growth and environmental impact: Ideology and academic discourse in Israel. Popul Environ 26: 41–60.

[43] Achiron-Frumkin T (2011) Report on the State of Nature 2010 The Maarag (LTER-Israel). Jerusalem: The Israel Academy of Sciences.

[44] Cooke SJ, Danylchuk AJ, Kaiser MJ, Rudd MA (2010) Is there a need for a '100 questions exercise' to enhance fisheries and aquatic conservation, policy, management and research? Lessons from a global 100 questions exercise on conservation of biodiversity. J Fish Biol 76: 2261–2286. 10.1111/j.1095-8649.2010.02666.x 20557662

