# Supplementary material for: Priority Questions and Horizon Scanning for Conservation: A Comparative Study
Source: PLoS One. 2016 Jan 27;11(1):e0145978. doi: 10.1371/journal.pone.0145978 (PMC4729468; doi:10.1371/journal.pone.0145978)
Supplement: S1 Appendix — A. List of organizations in Israel from which research questions and horizon scanning topics were submitted. B. List of organizations that participated in the priority questions and horizon scanning topic selection workshop in Israel. (DOCX) [file pone.0145978.s001.docx]

**S1 Appendix**

**A. List of organizations in Israel from which research questions and horizon scanning topics were submitted:**

The Israel Knesset Research and Information Center, The Israel Academy of Sciences and Humanities, The Hebrew University of Jerusalem, Tel-Aviv University, University of Haifa-Oranim, Bar-Ilan University, Ben-Gurion University of the Negev, Weizmann Institute of Science, The Technion, Heriot-Watt University (UK), Beit Berl College, Kinneret Academic College, Agricultural Research Organization – Volcani Center, The Jerusalem Institute for Israel Studies, Israel Oceanographic and Limnological Research Institute, The Ministry of Industry, Trade and Labor, The Ministry of Environmental Protection, Israel Nature and Parks Authority, Yarqon River Authority, The Jewish National Fund, The Society for the Protection of Nature in Israel, The Open Landscape Institute, The Israel Union for Environmental Defense, The Green Environment Fund, Friends of the Earth – Middle East, The Israel Society of Ecology and Environmental Sciences, Ramat Ha Nadiv Nature Park, Gidron Travels, Geo-Teva Environmental Consultants, The Israel Electric Corporation.

**B. List of organizations that participated in the priority questions and horizon scanning topic selection workshops in Israel**

The workshop included invited representatives from The Israel Ministry of Environmental Protection, The Israel Academy of Sciences and Humanities, The Hebrew University of Jerusalem, Tel Aviv University, University of Haifa, the Technion, Ben Gurion University in the Negev, the Weizmann Institute of Science, The Jewish National Fund-Keren Kayemeth LeIsrael (Israel's forestry agency), the Israel Nature and Parks Authority, the Open Landscape Institute, the Israel Society of Ecology and Environmental Sciences, the Ma’arag (Israel National Program for Ecosystem Assessment), The Yarqon Water Authority, The Society for the Protection of Nature in Israel and Friends of the Earth – Middle East.**S2 Appendix**

The definitions of horizon scanning in each of the horizon scanning in the projects included in this study. The definition, while sharing some attributes, differed among the projects conducted globally and compared here, which may have partly affected their different outcomes.

| **Definition of horizon scanning** **in UK project (Sutherland et al. 2008):** “The future novel or step changes in threats to, and opportunities for, biodiversity that might arise in the UK up to 2050, but that had not been important in the recent past.” |
| --- |
| **Definition of horizon scanning** **in global project from 2010 (Sutherland et al. 2010):** Horizon scanning identifies… “emerging issues in a given field sufficiently early to conduct research to inform policy and practice. Our group of horizon scanners… identified] fifteen nascent issues that could affect the conservation of biological diversity. [Horizon scanning is] the systematic search for incipient trends, opportunities and risks that may affect the probability of achieving management goals and objectives. The aim of horizon scanning is not to predict the future, but to identify emerging issues in sufficient time to initiate research and develop policy and practical responses. [Participants submitted] emergent issues that they felt were globally important or may have a local effect on species, ecosystems or regions of global interest.” |
| **Definition of horizon scanning** **in global project from 2011 (Sutherland et al. 2011):** “[The identification of] emerging issues that could have substantial impacts on the conservation of biological diversity… sufficiently early to encourage policy-relevant, practical research on those issues… the systematic search for incipient trends, opportunities and constraints that might affect the probability of achieving management goals and objectives. Explicit objectives of horizon scanning are to anticipate issues, accumulate data and knowledge about them, and thus inform crucial decisions. [The] aim was to identify technological advances, environmental changes, novel ecological interactions and changes in society that could have substantial impacts on the conservation of biological diversity… whether beneficial or detrimental.” |
| **Definition of horizon scanning** **in the Israel project (this paper):** “Future (10-25 years) issues and gaps in current research that are likely to have serious impacts on biodiversity conservation but do not currently receive sufficient scientific attention.” |

**Supporting information legend**

**S1 Appendix**

A. List of organizations in Israel from which research questions and horizon scanning topics were submitted:

B. List of organizations that participated in the priority questions and horizon scanning topic selection workshop in Israel:

**S2 Appendix**

The definitions of horizon scanning in each of the horizon scanning in the projects included in this study. The definition, while sharing some attributes, differed among the projects conducted globally and compared here, which may have partly affected their different outcomes.
