# Supplementary material for: Priority Questions and Horizon Scanning for Conservation: A Comparative Study
Source: PLoS One. 2016 Jan 27;11(1):e0145978. doi: 10.1371/journal.pone.0145978 (PMC4729468; doi:10.1371/journal.pone.0145978)
Supplement: S2 Appendix — The definition, while sharing some attributes, differed among the projects conducted globally and compared here, which may have partly affected their different outcomes. (DOCX) [file pone.0145978.s002.docx]

**S2 Appendix**

**The definitions of horizon scanning in the projects included in this study. The definition, while sharing some attributes, differed among the projects conducted globally and compared here, which may have partly affected their different outcomes**

| **Definition of horizon scanning** **in UK project (Sutherland et al. 2008):** “The future novel or step changes in threats to, and opportunities for, biodiversity that might arise in the UK up to 2050, but that had not been important in the recent past.” |
| --- |
| **Definition of horizon scanning** **in global project from 2010 (Sutherland et al. 2010):** Horizon scanning identifies… “emerging issues in a given field sufficiently early to conduct research to inform policy and practice. Our group of horizon scanners… identified] fifteen nascent issues that could affect the conservation of biological diversity. [Horizon scanning is] the systematic search for incipient trends, opportunities and risks that may affect the probability of achieving management goals and objectives. The aim of horizon scanning is not to predict the future, but to identify emerging issues in sufficient time to initiate research and develop policy and practical responses. [Participants submitted] emergent issues that they felt were globally important or may have a local effect on species, ecosystems or regions of global interest.” |
| **Definition of horizon scanning** **in global project from 2011 (Sutherland et al. 2011):** “[The identification of] emerging issues that could have substantial impacts on the conservation of biological diversity… sufficiently early to encourage policy-relevant, practical research on those issues… the systematic search for incipient trends, opportunities and constraints that might affect the probability of achieving management goals and objectives. Explicit objectives of horizon scanning are to anticipate issues, accumulate data and knowledge about them, and thus inform crucial decisions. [The] aim was to identify technological advances, environmental changes, novel ecological interactions and changes in society that could have substantial impacts on the conservation of biological diversity… whether beneficial or detrimental.” |
| **Definition of horizon scanning** **in the Israel project (this paper):** “Future (10-25 years) issues and gaps in current research that are likely to have serious impacts on biodiversity conservation but do not currently receive sufficient scientific attention.” |
